# Long-Term Swallowing Rehabilitation for Muscle-Specific Tyrosine Kinase Antibody-Positive Myasthenia Gravis: A Case Report

**DOI:** 10.7759/cureus.80544

**Published:** 2025-03-13

**Authors:** Yuki Oshima, Tomoki Nanto, Kazuki Eimoto, Yuki Uchiyama, Kazuhisa Domen

**Affiliations:** 1 Department of Rehabilitation, Hyogo Medical University Hospital, Nishinomiya, JPN; 2 Department of Speech-Language-Hearing Therapy, Faculty of Rehabilitation, Morinomiya University of Medical Sciences, Osaka, JPN; 3 Department of Rehabilitation Medicine, School of Medicine, Hyogo Medical University, Nishinomiya, JPN

**Keywords:** anti-musk antibody, dysphagia, myasthenia gravis, recurrent aspiration pneumonia, rehabilitation

## Abstract

Muscle-specific tyrosine kinase antibody-positive myasthenia gravis (MuSK-MG) often presents with severe dysphagia. However, effective strategies for swallowing rehabilitation in these patients have not been well established. We report a case of a MuSK-MG patient with severe dysphagia who underwent swallowing rehabilitation for approximately 12 months alongside drug therapy and showed improvement in swallowing function. The patient was a 67-year-old woman treated with high-dose intravenous methylprednisolone pulse therapy, high-dose intravenous immunoglobulin, and plasma exchange (PE) therapy. She required ventilator management and tracheotomy. The patient subsequently developed recurrent pneumonia, and rituximab was administered due to a poor response to previous treatments. Although the primary disease had apparently stabilized, the patient continued to experience severe dysphagia with silent aspiration, delayed swallowing reflexes, and pharyngeal residues. To address these issues, intensive and prolonged swallowing rehabilitation was implemented, including regular swallowing assessments, progressive muscle-strengthening exercises with controlled load levels, and interferential current stimulation to address sensory disturbances. Consequently, the patient successfully achieved oral intake without experiencing a myasthenic crisis or recurrence of aspiration pneumonia. These findings suggest that long-term swallowing rehabilitation, combined with appropriate treatment of the underlying disease, may effectively improve swallowing function in patients with MuSK-MG and persistent severe dysphagia.

## Introduction

Myasthenia gravis (MG) is an autoimmune disease characterized by neuromuscular junction dysfunction that leads to skeletal muscle weakness. MG is classified based on the presence or absence of serum autoantibodies. Approximately 80%-85% of MG cases are acetylcholine receptor antibody-positive myasthenia gravis (AChR-MG), 5%-8% of cases are muscle-specific tyrosine kinase antibody-positive myasthenia gravis (MuSK-MG), less than 1% of cases are low-density lipoprotein receptor-related protein 4-positive MG, and approximately 10% of cases are double seronegative MG [1]. MuSK-MG has a more acute onset, with dysphagia and bulbar palsy as the main symptoms, and it progresses more rapidly than AChR-MG [2,3]. Therefore, a videofluorographic swallowing study (VFSS), a radiographic test that evaluates swallowing function using contrast media, is important to objectively determine the appropriate treatment for MuSK-MG [4]. Symptoms are more severe in MuSK-MG than in AChR-MG, and half of the patients develop a myasthenic crisis during the course of the disease [5]. A myasthenic crisis is a potentially life-threatening complication of MG [A1] that requires mechanical ventilation due to respiratory muscle weakness [6]. Swallowing rehabilitation should be performed in parallel with the treatment and evaluation of underlying diseases. However, no study has reported on the long-term course of dysphagia, and the effectiveness of swallowing rehabilitation has not been clearly established. This case report describes the long-term course of severe dysphagia after a MuSK-MG myasthenic crisis and the effects of swallowing rehabilitation combined with drug therapy.

This article was presented at the 25th Annual Meeting of the Japan Society of Speech-Language-Hearing Therapists in Kobe, held on June 21, 2024.

## Case presentation

Participant

For this case report, written informed consent was obtained from the patient. A 67-year-old woman was diagnosed with MuSK-MG 31 years earlier and had been receiving outpatient treatment at our hospital. The patient originally had dysarthria and hypernasal speech. However, her Activities of Daily Living (ADL) status was 'independent,' and she was proficient in all household chores as a housewife. Four days before hospitalization, the patient experienced fatigue. She subsequently experienced difficulty swallowing her lunch and was admitted to the hospital with a diagnosis of myasthenic crisis. Rehabilitation by speech and physical therapists was started on the second day of hospitalization.

At the time of the initial evaluation, the patient was conscious and had no cognitive impairment. Her respiratory function required 1 L/min of oxygen, and her resting respiratory rate was 20 breaths/min with no notable abnormal sounds. The total functional independence measure (FIM) was 47 points (motor: 13 points; cognitive: 34 points). Oral facial movements were symmetrical. Tongue movement was restricted; however, her speech was sometimes intelligible. She had no fasciculation of the tongue, but she had tongue atrophy. The maximum tongue pressure, measured using a JMS tongue pressure device (JMS Co., Ltd., Hiroshima, Japan), was 4.6 kPa. Limited soft-palate elevation during phonation resulted in marked hypernasal speech. Pathological reflexes such as the snout, mandibular, and palmar reflexes were not observed. The patient exhibited hoarseness with asthenia. The maximum phonation time was 18 seconds. Swallowing screening tests revealed a Repetitive Saliva Swallowing Test score of 5, Modified Water Swallow Test score of 5, Water Swallow Test score of 2, and a Food Test score of 5. On the third day after admission, arterial blood gas analysis revealed carbon dioxide retention (PCO2: 62 mmHg). Owing to the high risk of aspiration due to the large amount of saliva accumulation, oral intubation was recommended, and the patient was managed with oral intubation.

**Figure 1:**
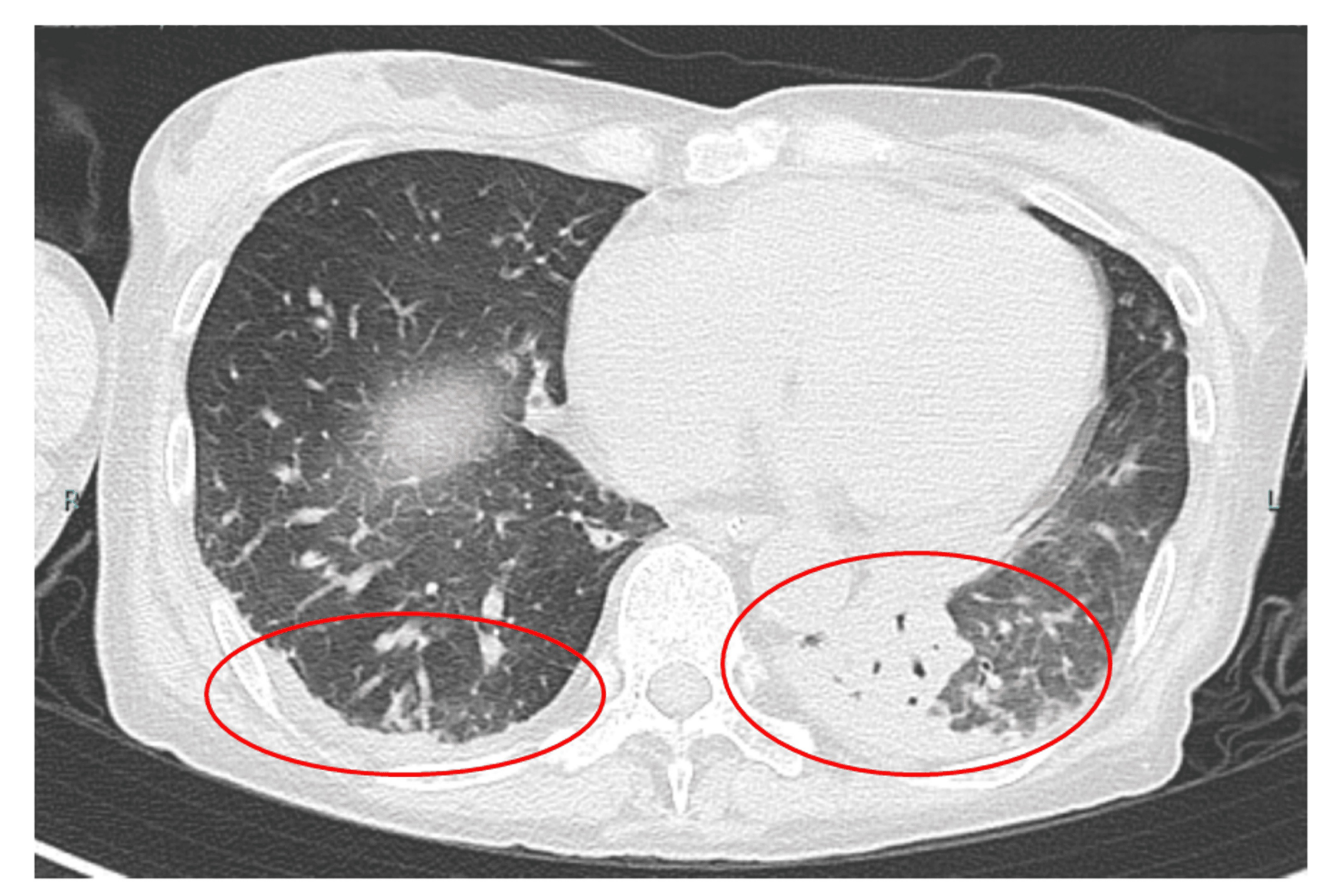
Coronal section of a chest CT showing bilateral lower lung wetting and increased bronchial translucency in the left lower lung.

The patient received high-dose intravenous methylprednisolone pulse therapy (IVMP) from days 3-5 to days 9-11, and plasma exchange therapy (PE) was administered six times beginning on day 15. She was extubated on day 14, but aspiration pneumonia was noted and reintubation was necessary due to poor oxygenation (Figure 1).

**Figure 2:**
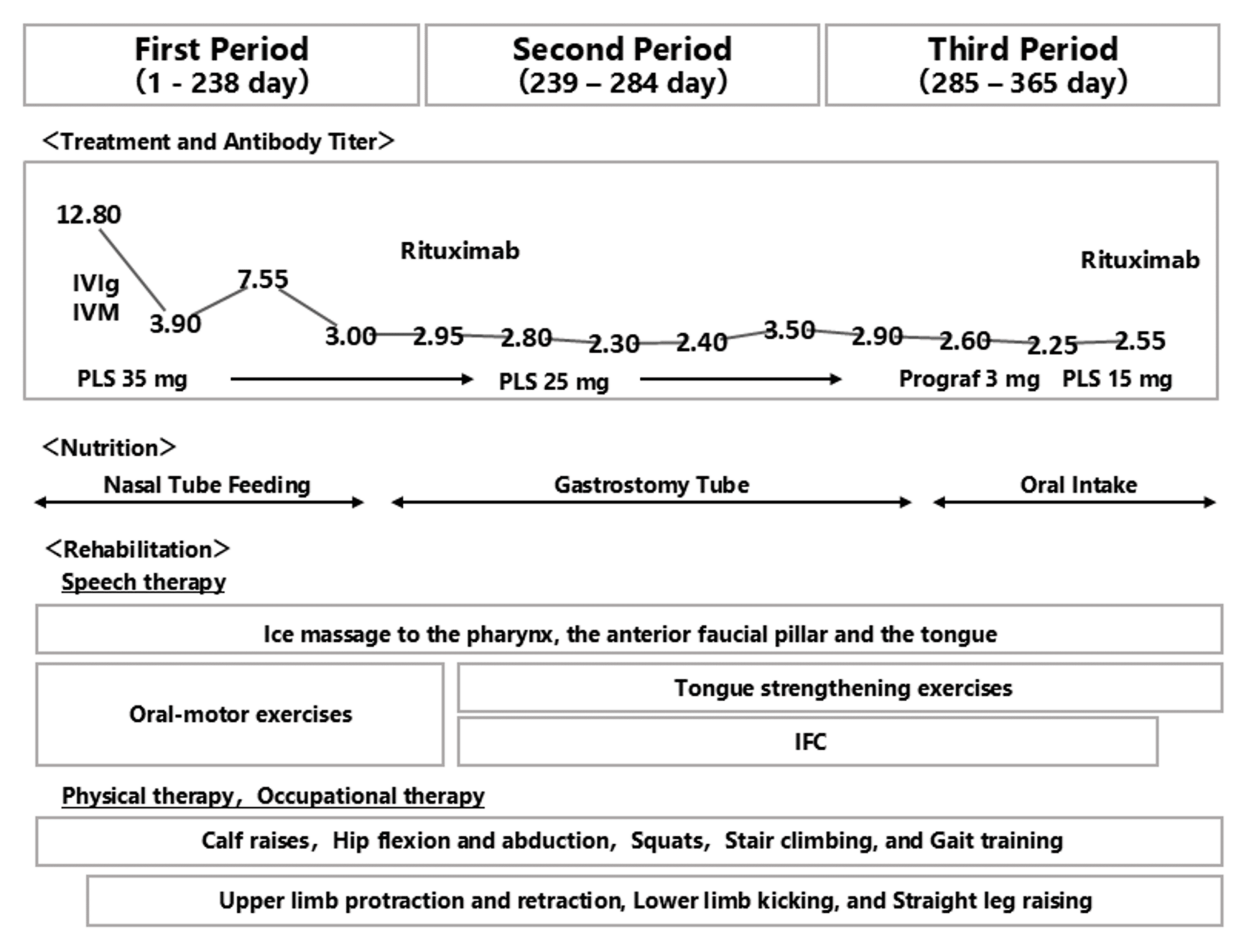
Timeline of the clinical course and treatment. The patient's course was divided into three phases based on the long-term nature of the disease. Changes in antibody titers during hospitalization are shown in the graph. The initial treatments consisted of IVIg, IVMP, and PE. Speech therapy included ice massage, followed by empty swallowing and orofacial exercises. Physical therapy comprised calf raising, hip flexion and abduction, squats, stair climbing, and gait training. Occupational therapy focused on upper limb protraction and retraction, lower limb kicking, and straight leg raising. During the second period, rituximab was administered, and nutrition was provided via gastrostomy. Speech therapy comprised tongue-strengthening exercises, chewing training, and IFC. The exercises and routines from the first phase were continued in physical and occupational therapy. During the third period, the patient was re-treated with rituximab. Swallowing function improved, and the patient was able to take oral nutrition exclusively. Speech therapy continued during the third period, alongside similar exercises and routines in physical and occupational therapy. IVIg: Intravenous immunoglobulin; IVMP: Intravenous methylprednisolone pulse therapy; PE: Plasma exchange therapy; IFC: Interferential current stimulation; PLS: Pulse steroid therapy. Note: Prograf (tacrolimus) is manufactured by Astellas Pharma Canada Inc., Markham, ON, Canada.

A tracheotomy was performed on day 18. Owing to the long-term hospitalization course, which extended to day 365, the entire course was divided into three phases. The overall course of the study is shown in Figure 2.

Rehabilitation progress

Phase 1 (Prevention Phase of Disuse Muscle Atrophy) on Days 1-238

Physical therapy included gait training for extremity muscle weakness and reduced respiratory function. On day 53, a fiberoptic endoscopic evaluation of swallowing was performed. It showed pharyngeal edema and a large amount of foamy saliva in the pharynx. Vocal cord mobility was good; however, soft-palate elevation was poor during swallowing and phonation. The whiteout was weak, and the cough reflex in response to epiglottic and arytenoid stimulation was absent. Swallowing-related muscle strength was significantly reduced, with a maximum tongue pressure of 5.7 kPa and jaw-opening force measured at 2.7 kg with a jaw-opening sthenometer (Livet Inc., Tokyo, Japan). The time to maintain maximum head lifting was 0 seconds. Swallowing rehabilitation focused on preventing the disuse of swallowing-related muscles and reducing pharyngeal sensation, including orofacial exercises and ice massage to the pharynx, anterior faucial pillar, and tongue. This procedure was followed by dry swallowing. Occupational therapy was initiated on day 61 of admission and consisted of upper limb protraction and retraction, lower limb kicking, and straight leg raising to prevent disuse of the upper and lower limbs and increase muscle strength. The patient underwent a videofluorographic swallowing study (VFSS) on day 120. The concentration of thickened water and the classification of meals followed the provisions of The Japanese Dysphagia Diet of 2021 (JDD2021) by the Japanese Society of Dysphagia Rehabilitation [7]. In the 30-degree reclined position, silent aspiration with 2 mL of moderately thick water was observed; however, a small amount of jelly equivalent to JDD2021 code 0 (Homogeneous, adhesive, cohesive, and firm jelly with consideration for adhesion, cohesion, and firmness) could be ingested without laryngeal penetration or pharyngeal residues. Oral intake training using jelly equivalent to JDD2021 code 0 was consequently initiated.

The patient received a single dose of rituximab on day 122 of admission after IVIg, IVMP, and PE were determined to be ineffective. However, oral intake was halted on day 125 due to fever and an elevated inflammatory response. The patient developed septic shock and was admitted to the ICU on day 140. From day 179 onward, rituximab was administered four times. The patient had recurrent pneumonia, which made continuing rehabilitation difficult. As the patient's swallowing function was not expected to improve in the short term, gastrostomy was performed on day 219. The patient required 1 L/min of oxygen during the day and mechanical ventilation (i.e., synchronized intermittent mandatory ventilation) at night with gradual weaning. The FIM score was 55 points (motor: 21 points; cognitive: 34 points).

Phase 2 (Active Training Phase; Days 239-284)

On day 239 of hospitalization, the patient underwent VFSS. In the 30-degree reclined position, aspiration did not occur with moderately thick water or with jelly equivalent to the JDD2021 code of 0. Some residues existed in the vallecula and pyriform sinuses after the jelly equivalent to JDD2021 code 0 was consumed; however, the residues disappeared with subsequent swallows. Silent aspiration was observed in the wheelchair-sitting position with 3 mL of moderately thick water (Figure 2). The next day, the patient was placed in a 30-degree reclining position and began training for oral intake of the jelly. As the patient’s symptoms subsided, continuous swallowing rehabilitation became feasible.

On the 240th day of hospitalization, swallowing-related muscle strength remained decreased, with a maximum tongue pressure of 7.8 kPa, jaw-opening force of 3.2 kg, and the time to maintain maximum head lifting of 8 seconds. Based on the VFSS and clinical evaluations, the patient’s symptoms included tongue muscle weakness, inadequate laryngeal elevation, reduced, and delayed swallowing reflex. For tongue muscle weakness and inadequate laryngeal elevation, tongue strengthening exercise was conducted using the Peco Panda device (JMS Co., Ltd., Hiroshima, Japan). Peco Panda is a tongue pressure training tool for strengthening the muscles of the tongue to improve swallowing and articulatory functions. It has six levels of strength: super-soft (SS), soft (S), medium-soft (MS), medium (M), medium-hard (MH), and hard (H). The color and load of the press were set for each type: SS, blue, 5 kPa; S, pink, 10 kPa; MS, purple, 15 kPa; M, green, 20 kPa; MH, orange, 25 kPa; and H, yellow, 30 kPa. Interferential current stimulation (IFC) was implemented to improve delayed initiation of the swallowing reflex. IFC was conducted using Gentle Stim stimulator (Caleido Co., Ltd., Kanagawa, Japan) with a beat frequency of 50 Hz and a carrier frequency of 2000 Hz. Stimulation electrodes were attached to the skin on both sides of the anterior neck, and the stimulation intensity was set at one level below the intensity at which the patient could feel stimulation [8]. The stimulation time was 30 min/day, 5 days/week, combined with swallowing rehabilitation.

The maximum tongue pressure and jaw-opening force were repeatedly measured as objective indicators of exercise load. However, obtaining stable measurements was difficult because the patient complained of fatigue and discomfort. Therefore, the Modified Borg Scale, an indicator of perceived exertion, was used as the standard exercise load. Tongue fatigue is frequently assessed during tongue elevation. At level 4 (i.e., “somewhat strong”) on the Borg scale, the patient reported numbness of the tongue and jaw. At level 5 (i.e., “strong”), the numbness and fatigue were pronounced and persistent. Level 4 was consequently set as the upper limit for training cessation. Specific measures were as follows: the day's fatigue was assessed before intervention; if the Modified Borg Scale score was 4 or higher, only ice massage was administered, followed by dry swallowing. Strength training was terminated when tongue fatigue reached level 4. Furthermore, consecutive sessions of physical therapy and speech therapy were avoided. The initial load for the Peco Panda device was 15 repetitions (5 reps × 3 sets) at the SS intensity, reaching level 4 on the Borg scale. The intensity and frequency of training were gradually increased, based on the cessation criteria. After 112 days of training, the patient performed 120 repetitions (10 repetitions × 12 sets) at the M intensity.

On the 267th day, the patient underwent VFSS. In the wheelchair-sitting position, silent aspiration was observed with 3 mL of mildly thick water and jelly equivalent to JDD2021 code 0. Porridge and meatballs were consumable without residue or aspiration. Therefore, a diet equivalent to JDD2021 code 2 (Homogeneous, smooth, non-sticky, and easy to clump together) was started on day 273 of hospitalization. Physical therapy included calf raises, hip flexion and abduction, squats, stair climbing, and walking exercises to prevent disuse of the upper and lower limbs and to increase muscle strength. Occupational therapy consisted of upper limb protraction and retraction, lower limb kicking, and straight leg raising. The FIM score improved to 79 points (motor: 45 points; cognitive: 34 points). Respiratory function also improved, and the patient was weaned off the ventilator on day 261 of hospitalization.

Phase 3 (Oral Intake Expansion Phase; Days 285-365)

On the 288th day, the cannula was changed to a cuffed and fenestrated double-lumen type. On the 285th day of hospitalization, in the wheelchair-sitting position, no aspiration of 3 mL of mildly thick water occurred; however, laryngeal penetration was observed with 5 mL of liquid. Rice and bread were consumed without aspiration or residue, and with additional swallows. Laryngeal penetration was observed with dual-phase foods, such as vegetables, and 3 mL of thinly thickened water or 20 mL of mildly thick water. The diet was upgraded to JDD2021 code 3 (Shaped, but easily crushed; easily formed into a food mass and transported; easy to swallow without flaking or breaking apart in the pharynx). The FIM score improved to 111 points (motor: 77 points; cognitive: 34 points).

The patient was able to breathe comfortably with a speech valve and experienced reduced sputum production, thereby enabling stable food intake. The cannula was consequently removed on the 299th day. Swallowing-related muscle strength on day 330 of hospitalization showed improvement, with a maximum tongue pressure of 13.9 kPa, jaw-mouth opening force of 6.7 kg, and time to maintain maximum head lifting of 21 seconds. On the 330th day, the patient underwent VFSS. In the wheelchair-sitting position, laryngeal penetration with 20 mL of mildly thick water had disappeared. However, laryngeal penetration was observed with 3 mL of liquid and vegetable dual-phase food. Silent aspiration occurred with 20 mL of liquid.

For safety, mildly thick water was maintained for liquids, and she was allowed to consume a diet equivalent to JDD2021 code 4 (No hardness, disjointedness, or stickiness; softness that can be cut with chopsticks or a spoon). The patient received four doses of rituximab starting on the 340th day, but no improvement occurred in swallowing-related muscle strength or swallowing function. The patient was discharged home on the 365th day of hospitalization (Table 1).

**Table 1:** Changes in swallowing-related muscle strength, swallowing function, and physical function. RSST: Repetitive saliva swallowing test; FIM: Functional Independence Measure. Note: 'N/A' indicates that no established reference range is available.

Period	Days 1-2	Days 57-66	Days 234-240	Days 325-330	Days 355-358	Reference Range
Maximum tongue pressure (kPa)	4.6	5.7	7.8	13.7	13.9	38.0 ± 9.0
Jaw-opening force (kg)	-	2.7	3.2	6.7	6	5.9 ± 1.6
RSST (count)	5	5	7	6	6	More than 3 times
Time to maintain maximum head raising (seconds)	-	0	8	21	21	N/A
Weight (kg)	37.1	39.7	38	36.3	35.3	N/A
BMI (kg/m²)	17.8	19.1	18.3	17.5	17	18.5-25.0
FIM (points)	28	47	62	81	99	N/A
FIM motor (points)	13	13	28	47	75	N/A
FIM cognition (points)	15	34	34	34	34	N/A

Quantitative evaluation using a VFSS study

The patient underwent VFSS six times during hospitalization. The analysis focused on swallowing dynamics during one swallow of 3 mL of moderately thick water in a sitting position without compensatory strategies. The analysis included the 3rd, 4th, 5th, and 6th tests. The sample of moderately thick water was prepared by diluting 200 mL of 40% barium water (Baritop P, 99% w/w; Kaigen Pharma Co., Osaka, Japan) with a xanthan gum-based thickener (Tsururinko Quickly, Morinaga Milk Industry Co., Tokyo, Japan). The viscosity was confirmed to be “moderately thick” (150-300 mPa·s), according to JDD2021 standards [7], using a cone-plate type viscometer (JOVI; Nutri Co., Mie, Japan).

The analysis parameters included the normalized residue ratio scale (NRRS) for evaluating pharyngeal residues. The residue in the valleculae was evaluated using NRRS for the valleculae (NRRSv) and that in the piriform sinuses using NRRS for the piriform sinus (NRRSp) [9]. The stage transition duration (STD) was analyzed as an indicator of the swallowing reflex, defined as the time from when the thickened water reached the mandibular ramus to when the hyoid bone began its rapid anterior movement [10]. The hyoid bone movement was analyzed for the total, upward, and forward movement distances [11]. Analysis was conducted using ImageJ (National Institutes of Health, Bethesda, MD, USA).

The results of this analysis are presented in Table 1. The NRRS, an indicator of pharyngeal residue, showed that NRRSv decreased from 1.28 to 0, and NRRSp decreased from 0.01 to 0, indicating the disappearance of residue. The STD, an indicator of the swallowing reflex, shortened from 0.60 seconds to 0.45 seconds. The hyoid movement distance improved from 5.55 mm to 13.2 mm (Table 2).

**Table 2:** VFSS analysis results. VFSS: Videofluorographic swallowing study; NRRSv: NRRS for the valleculae; NRRSp: NRRS for the piriform sinus; STD: stage transition duration. N/A indicates that no established reference range is available.

Test	1st (day 239)	2nd (day 267)	3rd (day 285)	4th (day 330)	Reference Range
NRRSv	1.28	0.05	0.21	0	N/A
NRRSp	0.01	0	0.01	0	N/A
STD (seconds)	0.6	0.83	0.73	0.45	N/A
Distance movement of the hyoid (mm)	5.55	6.81	8.14	13.2	N/A
Distance of the superior movement of the hyoid (mm)	4.9	5.77	7.5	8.12	N/A
Distance of the anterior movement of the hyoid (mm)	2.61	3.63	3.15	10.42	N/A

## Discussion

We conducted long-term swallowing rehabilitation in parallel with pharmacotherapy in a patient with MuSK-MG who presented with severe dysphagia. As a result, dysphagia persisted; however, the swallowing function eventually improved, and the patient was discharged. To the best of our knowledge, this report is the first to demonstrate the effectiveness of long-term swallowing rehabilitation in a patient with MuSK-MG.

Characteristics of dysphagia

Only three reports detail the characteristics of dysphagia in patients with MuSK-MG [6,12,13]. The features of dysphagia in MuSK-MG include inadequate elevation of the soft palate, pharyngeal constriction failure, incomplete closure of the epiglottis, incomplete opening of the esophageal inlet, and pharyngeal residue with possible laryngeal penetration and aspiration. In this patient, similar to previous reports, inadequate elevation of the soft palate, pharyngeal constriction failure, incomplete closure of the epiglottis, pharyngeal residue, laryngeal penetration, and silent aspiration were observed. MuSK antibody levels are an indicator of MG disease severity, and MuSK antibody levels correlate with severity [14,15]. The patient's MuSK antibody levels remained relatively low, at approximately 3.00 nmol/L (normal value: <0.02 nmol/L), from the 90th day of hospitalization (Figure 1). Based on these observations, we conclude that the patient’s dysphagia was significantly influenced by MG and by both the decline in swallowing-related muscle strength and sensory impairment due to disuse.

Swallowing rehabilitation

Prolonged fasting periods can exacerbate the decline in swallowing function and increase the risk of silent aspiration, thereby extending recovery time [16]. The decline in swallowing-related muscle strength could be attributed to prolonged mechanical ventilation and more than 200 days of fasting due to repeated aspiration pneumonia, leading to disuse atrophy. Tongue resistance training using the Peco Panda device was used to improve the decline in swallowing-related muscle strength. Muscle weakness in MG is generally exacerbated by exercise and repeated muscle use [17]. To prevent muscle weakness from overuse, the patient must be cautious about fatigue during swallowing training. However, no report has addressed the appropriate load for swallowing training. Setting the cessation criteria based on fatigue tests of swallowing-related muscle groups (e.g., quantitative MG score, jaw-mouth opening strength, maximum tongue pressure, and time to maintain maximum head lifting) was deemed desirable. However, due to the patient's complaints of burden, pain, difficulty in implementation, and variability in measurements, setting the cessation criteria was challenging to perform. Therefore, the Modified Borg Scale, a subjective measure of exercise intensity, was used. The patient's cognitive function was intact, making the Modified Borg Scale a reliable and useful tool for assessing subjective exercise intensity. Low-to-moderate-intensity training for MG management has short-term and long-term benefits [18]. Additionally, the patient needed to consider the decreased cardiac function due to prolonged bed rest and reduced respiratory function. Hence, the exercise load should be moderate or low.

Furthermore, during muscle strengthening training, the onset of tongue or jaw numbness occurred at level 4 ('somewhat strong'), and at level 5 ('strong'), the numbness became more pronounced and fatigue persisted. Therefore, setting the cessation criterion at level 4 ('somewhat strong') was deemed appropriate. This approach allowed for a gradual increase in exercise load without adverse events such as crisis recurrence or aspiration pneumonia. Setting cessation criteria, even for swallowing training, is crucial in patients at risk of crisis recurrence. Additionally, sensory impairments such as delayed initiation of the swallowing reflex and silent aspiration were also considered secondary factors beyond the progression of MG. The larger the accumulation of secretions, the higher the sensory threshold in the pharyngolaryngeal region, reducing sensory perception and airway protective reflexes [19]. The patient exhibited significant saliva aspiration and pharyngeal secretion accumulation, suggesting a reduction in peripheral sensory input to the laryngeal mucosa. Furthermore, physical irritation caused by long-term cannula or nasogastric tube placement may have contributed to the increased sensory threshold in the pharyngolaryngeal region.

Training for decreased sensitivity in the pharyngolaryngeal region: To prevent hyposensitization of the pharynx, ice massage of the pharynx, anterior faucial pillar, and tongue was administered, followed by dry swallowing. The pharynx, anterior faucial pillar, and tongue have immediate effects on the swallowing reflex and significantly shorten the latency time required to trigger the swallowing reflex [20]. The IFC was also introduced to stimulate pharyngeal sensation, and its mechanism of action may be that interference wave stimulation applied to the neck enhances afferent impulses transmitted from the pharynx and larynx to the brainstem via the superior laryngeal nerve, thereby promoting the induction of the swallowing reflex [8]. IFC for approximately 2 months shortened the STD from 0.6 seconds to 0.45 seconds. These results suggested that ice massage to the pharynx, anterior faucial pillar, tongue, and IFC may be effective in the treatment of sensory loss secondary to nasotracheal tube feeding and cannulation.

This case report had several limitations. First, because this was a case report, the results cannot be generalized. Second, whether the improvement in swallowing function was due to pharmacotherapy or rehabilitation could not be determined. However, long-term rehabilitation combined with pharmacotherapy may improve swallowing function. No reports exist on the effects of long-term swallowing rehabilitation in patients with MuSK-MG. Therefore, more cases need to be studied in the future.

## Conclusions

This study is the first to report on the long-term rehabilitation of a patient with severe dysphagia following a MuSK-MG myasthenic crisis. The patient underwent a rehabilitation program involving regular swallowing assessments, progressive muscle strengthening with controlled load levels, and interferential current stimulation (IFC) to address sensory disturbances. These interventions led to significant improvements in swallowing-related muscle strength and function. Additionally, using the Modified Borg Scale to manage exercise intensity enabled gradual progress while preventing myasthenic crisis recurrence or aspiration pneumonia. These findings suggest that swallowing rehabilitation, individualized exercise load management, and sensory stimulation can effectively improve swallowing function in patients with persistent severe dysphagia after a myasthenic crisis.
